# Plasma exchange and double plasma molecular adsorption system combined with continuous renal replacement therapy for hepatitis B-related liver failure complicated by stage 3 acute kidney injury: a case report

**DOI:** 10.3389/fmed.2026.1945617

**Published:** 2026-09-21

**Authors:** Ding Zheng, Ran Li, Xiaobin Li, Zhaoxuan Peng, Jurong Liu, Xiaodong Wang, Mengyun Peng, Xiaoning Zhu, Jing Wang

**Affiliations:** 1 Hepatobiliary Department, The Affiliated Traditional Chinese Medicine Hospital, Southwest Medical University, Luzhou, China; 2 Sichuan Clinical Research Center for Traditional Chinese Medicine of Liver Diseases, The Affiliated Traditional Chinese Medicine Hospital, Southwest Medical University, Luzhou, China; 3 Intensive Care Unit, The Affiliated Traditional Chinese Medicine Hospital, Southwest Medical University, Luzhou, China

**Keywords:** acute kidney injury, continuous renal replacement therapy, double plasma molecular adsorption system, hepatitis B-related liver failure, plasma exchange

## Abstract

**Background:**

Hepatitis B virus (HBV)-related liver failure can progress rapidly to neurological and renal dysfunction. In this setting, declining aminotransferase levels may be misleading if hepatic synthetic function and extrahepatic organ function continue to deteriorate.

**Case presentation:**

A 27-year-old woman presented with severe fatigue, dark urine, and jaundice. Laboratory testing showed marked hepatocellular injury, coagulopathy, HBV infection markers, and an HBV DNA level of 3.57 × 10⁶ copies/mL. During hospitalization, serum aminotransferase levels declined substantially; however, bilirubin and coagulation abnormalities worsened, serum creatinine increased from 96 μmol/L on day 1 to 825 μmol/L on day 4, and the patient developed hypersomnia and impaired consciousness. The renal deterioration met criteria for stage 3 acute kidney injury. Following multidisciplinary assessment, plasma exchange combined with a double plasma molecular adsorption system was used for liver-directed extracorporeal support, while continuous renal replacement therapy was initiated primarily for severe acute kidney injury and metabolic support. Consciousness, coagulation, liver function, and renal function subsequently improved. The patient left the intensive care unit on day 7, was discharged on day 24, and maintained normal liver and kidney function during follow-up.

**Conclusion:**

This case highlights that declining aminotransferase levels do not necessarily indicate clinical recovery in rapidly progressive HBV-related liver failure. Serial assessment of hepatic synthetic function, neurological status, and renal function identified continued multiorgan deterioration and guided timely, indication-based extracorporeal support. The clinical value of this case lies in the decision-making process for coordinated liver and renal support rather than in the novelty of the individual treatment modalities.

## 1 Introduction

Hepatitis B virus (HBV)-related liver failure is a critical syndrome characterised by acute hepatic decompensation and frequently leads to multiorgan failure, with a high short-term mortality rate (1). Among the various complications, Acute kidney injury (AKI) is a frequently observed and prognostically important complication in patients with HBV-related liver failure (2). The development of AKI significantly worsens patient prognosis, and higher AKI stages are associated with substantially increased short-term mortality (3). Although plasma exchange, plasma adsorption, and continuous renal replacement therapy are established extracorporeal interventions, a practical challenge is determining when organ support should be escalated and which modality should be selected for each component of multiorgan dysfunction. Here, We report a case of HBV-related liver failure presenting with a marked decrease in aminotransferase levels, along with progressive coagulopathy, hyperbilirubinemia, neurological deterioration, and stage 3 acute kidney injury. This case illustrates the importance of longitudinal multiorgan assessment and indication-based coordination of extracorporeal liver and renal support.

## 2 Case presentation

A 27-year-old woman presented to a local hospital with profound fatigue and dark urine for 2 days. She had donated blood several times, and had no known history of hepatitis B or hepatitis C infection. Her close relatives had no history of chronic viral hepatitis. On admission, her blood pressure was 101/62 mmHg, and her pulse rate was 72 beats/min. Physical examination showed jaundice of the skin and sclerae, with right upper abdominal tenderness and rebound tenderness. Laboratory tests revealed severe hepatocellular injury and coagulopathy (Table 1). Hepatitis B surface antigen was positive, and the HBV DNA level was 3.57 × 10^6^ copies/mL (Table 2).

**Table 1:** Laboratory results during hospitalization and follow-up.

Time point	PT (s)	INR	ALB/ (g/L)	TBIL (*μ*mol/L)	DBIL (μmol/L)	ALT (U/L)	AST (U/L)	CR (μmol/L)	BUN (mmol/L)	NH_3_ (μmol/L)
Day 1	20.9	1.77	43.2	23.1	13.5	9,931	15,454	96	5.00	84.40
Day 3	42.2	4.35	33.3	143.7	125.9	7,187	4,055	645	19.60	NA
Day 4	34.5	3.35	33.2	165.3	140.9	4,295	1,327	825	21.66	NA
Day 5	26.7	2.41	25.2	165.5	139.1	2,723	478	452	13.92	NA
Day 6	18.6	1.52	30.9	195.4	164	2,068	364	237	7.60	NA
Day 7	15.7	1.23	29.6	154.2	123.8	1,465	239	259	5.98	50.00
Day 8	15.2	1.19	30.9	105.5	92.8	598	86	358	10.30	NA
Day 9	14.7	1.13	27.7	101.7	92.9	498	77	176	12.87	NA
Day 10	14.4	1.11	32.4	103.4	91.6	373	54	164	9.50	27.10
Day 11	13.9	1.06	31.4	88.9	80.6	310	47	148	5.30	NA
Day 12	13.6	1.02	30.2	78.4	66	230	51	293	3.50	NA
Day 13	NA	NA	32.8	66.1	54.2	169	63	279	7.42	NA
Day 15	13.9	1.05	37.6	53.1	39.3	130	46	168	9.17	NA
Day 20	12.8	0.95	47.2	44.4	29.1	79	46	97	7.72	NA
Day 24	12.5	0.92	47.4	38.2	23.2	60	49	76	9.41	NA
Day 90	NA	NA	42.9	15.1	6.4	22	20	55	3.40	NA

PT, prothrombin time; INR, international normalized ratio; ALB, albumin; TBIL, total bilirubin; DBIL, direct bilirubin; ALT, alanine aminotransferase; AST, aspartate aminotransferase; Cr, creatinine; BUN, blood urea nitrogen; NH3, ammonia; NA, not available.

**Table 2:** Hepatitis B serology and HBV DNA levels.

Time point	HBsAg	Anti-HBs	HBeAg	Anti -HBe	Anti -HBc	HBV-DNA
Day 1	+	+	+	-	-	3.57 × 10^6^ Copies/mL
Day 15	-	+	-	+	+	<2.0 × 10^2^ Copies/mL
Day 20	NA	NA	NA	NA	NA	<1.0 × 10^1^ IU/mL
Day 90	-	+	-	+	+	

Symbols: +, positive; -, negative; NA, not available.

On day 3, serum ALT and AST levels had decreased substantially; however, this biochemical change was accompanied by continued deterioration in other measures of organ function. Total bilirubin increased to 143.7 μmol/L, INR increased to 4.35, and serum creatinine increased from 96 μmol/L on day 1 to 645 μmol/L. Abdominal contrast-enhanced MRI showed reduced volume of the left hepatic lobe and fatty liver (Figure 1A, B).

**Figure 1:**
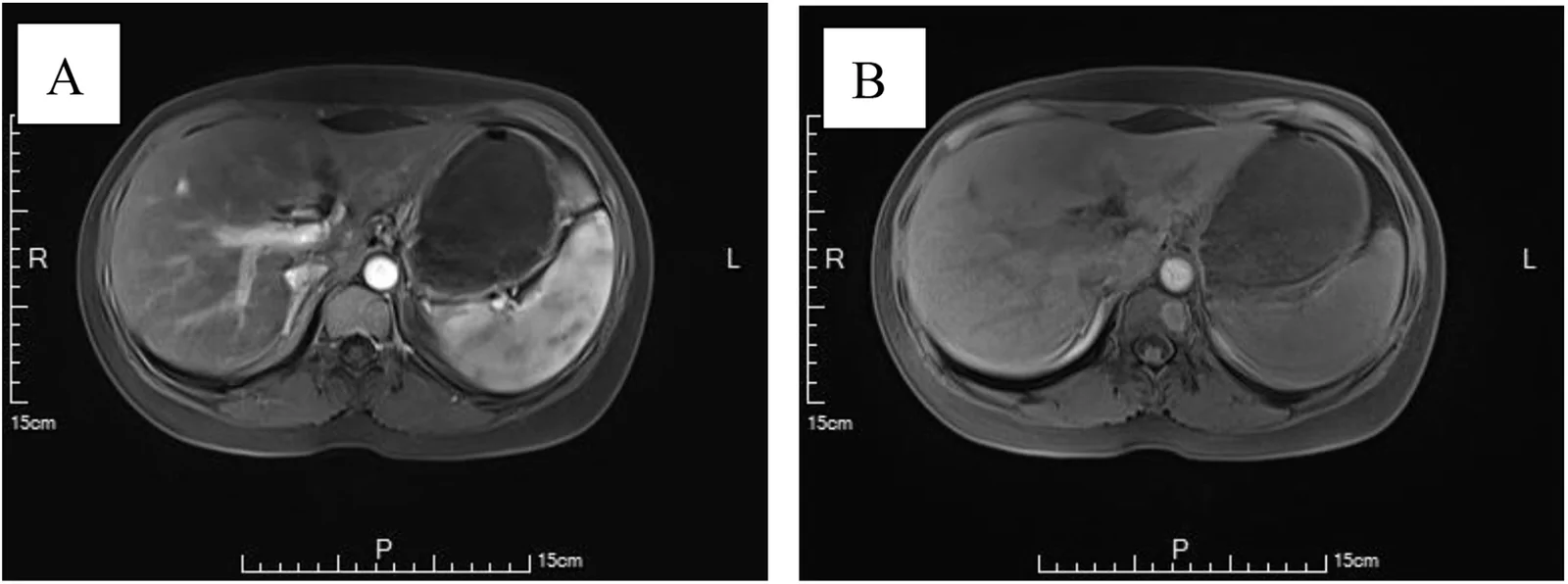
Abdominal contrast-enhanced MRI findings. The left hepatic lobe was reduced in size on the plain scan **(A)** and arterial phase **(B)**.

On day 4, serum creatinine further increased to 825 μmol/L and total bilirubin to 165.3 μmol/L. The patient developed hypersomnia and a progressive reduction in consciousness, consistent with hepatic encephalopathy in the context of worsening liver failure. Because a formal West Haven grade or Glasgow Coma Scale score was not documented in the available case data, a retrospective neurological grade was not assigned. According to KDIGO criteria, the rapid increase in serum creatinine and subsequent requirement for renal replacement therapy were consistent with stage 3 acute kidney injury. Following multidisciplinary assessment, the patient was transferred to the intensive care unit for organ-support treatment.

PE combined with DPMAS was initiated as liver-directed extracorporeal support. PE was used to remove circulating substances while replacing plasma components, including coagulation factors and albumin, whereas DPMAS provided additional adsorption of bilirubin, bile acids, and other circulating toxic substances. For PE plus DPMAS treatment, a double-lumen catheter was inserted into the internal jugular vein to establish extracorporeal vascular access. Plasma was separated and processed using an HA330-II hemoperfusion cartridge and a BS330 plasma bilirubin adsorber. Approximately 1,400 mL of plasma was processed and replaced with approximately 1,400 mL of fresh plasma. One PE/DPMAS session was performed. CRRT was subsequently performed using an HF1200 hemofilter, with two treatment sessions completed. The PE + DPMAS and CRRT treatments were performed sequentially, not simultaneously, using the same double-lumen central venous catheter placed in the internal jugular vein. Following the completion of the PE/DPMAS session, CRRT was initiated on the same vascular access. The CRRT prescription was as follows: HF1200 hemofilter, continuous veno-venous hemodiafiltration (CVVHDF) mode, blood flow rate of 150–180 mL/min, replacement fluid flow rate of 2,000–2,500 mL/h, and dialysate flow rate of 1,500–2000mL/h. Regional citrate anticoagulation was used. Each of the two CRRT sessions lasted approximately 24 h.

On day 7, the patient's consciousness, liver function, renal function, and coagulation function had markedly improved, and she was transferred from the intensive care unit back to the general department for further treatment. However,on the 8th day, there was a brief rebound in creatinine levels. On day 24, her transaminase, bilirubin, and creatinine levels had almost returned to normal (Table 1). Hepatitis B surface antigen had become negative (Table 2). Liver biopsy showed chronic active hepatitis with mild active inflammation (Figure 2). On day 90, liver and kidney function remained normal, and hepatitis B surface antibody was positive (Tables 1, 2).

**Figure 2:**
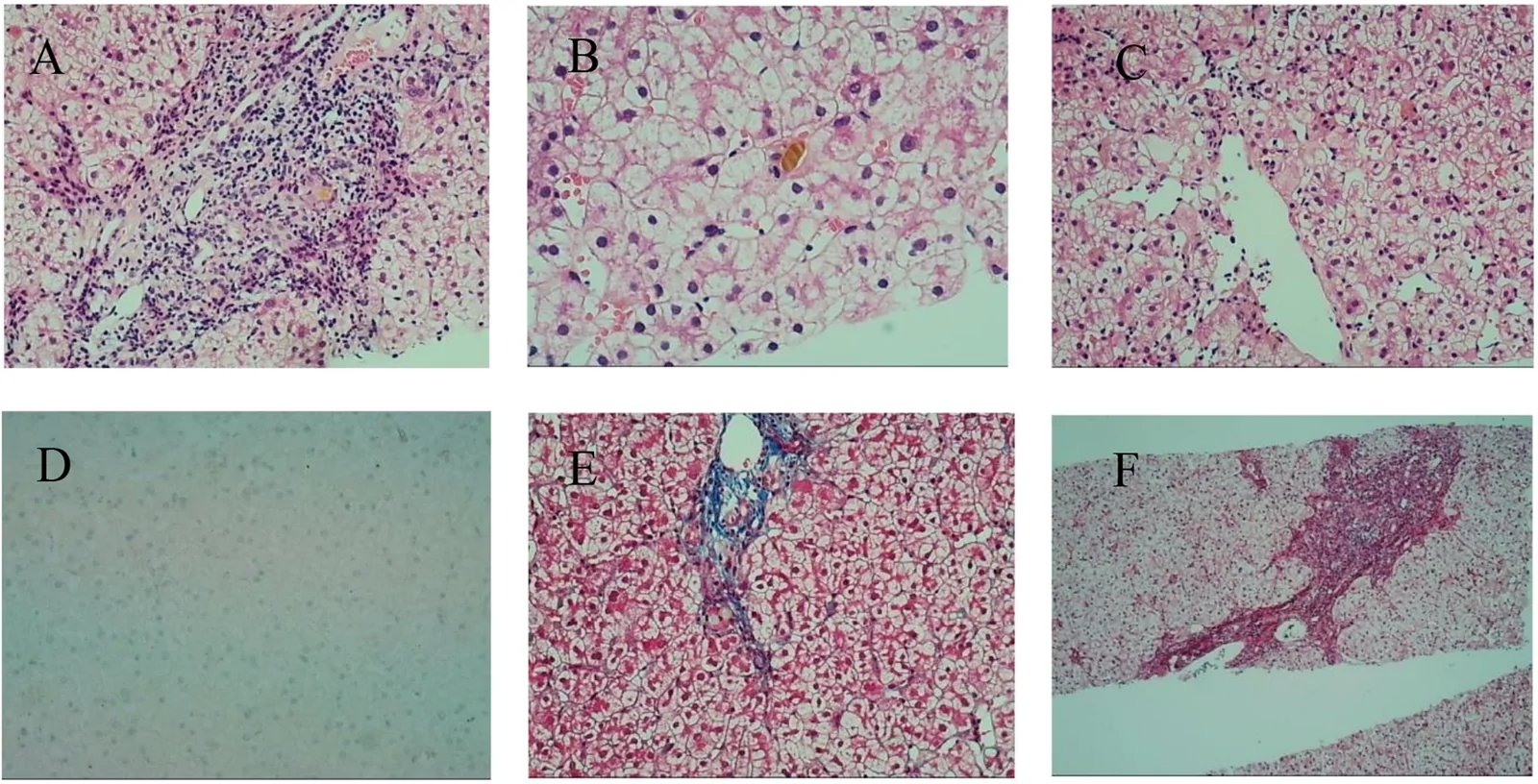
Liver biopsy pathological findings. **(A)** Portal and interface inflammation with bile duct embolus. **(B)** Capillary bile duct dilatation, cholestasis, and bile embolus. **(C)** Necrosis around the central vein. **(D)** Negative HBsAg staining. **(E,F)** Fibrous expansion in the portal area with stellate fibrosis and a few short fibrous septa between lobules.

## 3 Discussion

The principal clinical contribution of this case is the demonstration of a decision-making process in which longitudinal, multi-organ function assessment guided the sequential and coordinated use of multimodal extracorporeal therapies in a patient with HBV-related liver failure and stage 3 AKI. The marked discordance between declining aminotransferase levels and worsening synthetic function, coagulopathy, neurological status, and renal function signaled progression to multi-organ failure, rather than recovery. Recognizing this trajectory prompted a multidisciplinary reassessment that led to an indication-based strategy: PE/DPMAS for liver-directed support and CRRT primarily for severe AKI and metabolic management. This case illustrates how dynamic, multi-organ assessment, rather than reliance on any single laboratory parameter, is critical for the timely escalation and appropriate selection of complementary organ-support modalities.

This patient had no known history of chronic viral hepatitis and no family history of chronic viral hepatitis. During this period of hospitalization, she tested positive for hepatitis B surface antigen and exhibited a high level of HBV DNA. Other potential causes of hepatitis and liver injury, such as additional viral hepatitis, alcohol-related liver damage, metabolic-associated fatty liver disease, autoimmune hepatic conditions, and drug-induced hepatotoxicity, were thoroughly considered and subsequently excluded based on comprehensive history-taking and laboratory assessment. These findings are compatible with a recent HBV infection but do not, by themselves, definitively distinguish primary acute hepatitis B from previously unrecognized chronic HBV infection with acute exacerbation. In particular, the available case data did not include a quantitative IgM anti-HBc result or documented previous HBsAg-negative testing that could establish recent acquisition of HBV with greater confidence. At the time the patient's clinical condition stabilized (with normalized vital signs and controlled encephalopathy), we were faced with a fundamental management decision: whether this was Acute Hepatitis B (AVH-B) with spontaneous clearance potential, or Acute-on-Chronic Hepatitis B (CHB-AE) with underlying cirrhosis/fibrosis. These two entities dictate completely different long-term strategies regarding antiviral therapy duration, discontinuation timing, and hepatocellular carcinoma (HCC) surveillance frequency. Serum markers alone (HBV-DNA, IgM anti-HBc) were unable to definitively differentiate between the two, as high-level viremia and positive IgM can occur in both scenarios. Therefore, liver histology was pursued as the gold standard to guide long-term prognosis, rather than to evaluate the immediate recovery from acute failure.

AKI is a relatively common complication of hepatitis B-related liver failure. In a large-scale retrospective cohort study that enrolled 86,204 patients with HBV infection without baseline cirrhosis or hepatocellular carcinoma, 4,407 (5.1%) patients were found to have experienced AKI (3). The development of severe AKI necessitates rapid multidisciplinary intervention. While CRRT was initiated in this patient primarily for stage 3 AKI, its potential benefits in the context of liver failure extend beyond renal replacement. Emerging evidence and guidelines suggest that CRRT can be employed to manage hyperammonemia and hepatic encephalopathy, even in the absence of conventional renal replacement therapy indications (3–5). In this case, CRRT contributed to the control of fluid balance, electrolytes, and acid-base status, and may have also assisted in managing the metabolic consequences of liver failure. This highlights that the clinical effects of CRRT are multi-faceted, although its principal indication in this patient remained renal support.

Hepatitis B-related liver failure is a critical illness with an acute onset, rapid progression, and high mortality (6). Due to the clinical progression, which may advance rapidly, a significant number of patients loses the opportunity for timely liver transplantation. With advancements in intensive care and emergency liver transplantation, the mortality rate of Hepatitis B-related liver failure has decreased (7). Artificial liver support is an important supportive strategy in treating Hepatitis B-related liver failure. Commonly utilized systems comprise the Molecular Adsorbent Recirculating System (MARS) and the Plasma Separation and Adsorption System (SEPAD) (8, 9). In China, PE is a common non-bioartificial liver support therapy that removes toxins related to liver failure and supplies active substances, including coagulation factors and albumin. However, PE alone has a limited ability to clear inflammatory mediators, creatinine, and other small-molecule toxins. DPMAS connects a bilirubin adsorber and resin perfusion device to the plasma circuit. It can adsorb bilirubin and bile acids and may also remove endotoxin and inflammatory mediators while reducing plasma consumption (10).

In this case, the treatment effects of PE, DPMAS, and CRRT were complementary. DPMAS may have contributed to bilirubin, bile acid, endotoxin, and inflammatory mediator removal. PE may have compensated for the loss of beneficial plasma components, such as coagulation factors and growth factors, and may have supported liver regeneration (11). In this patient, the combination was therefore used as an individualized liver-support strategy addressing both toxin removal and plasma-component replacement. Nevertheless, because PE and DPMAS were administered together, the clinical contribution of each component cannot be separated in this single case.

The principal clinical lesson from this case is the value of systematic, longitudinal assessment of multiple organ systems—rather than reliance on a single laboratory trend—in guiding the timely escalation and appropriate selection of complementary extracorporeal therapies. In this patient, the discordant improvement in transaminase levels alongside worsening synthetic, neurological, and renal function prompted a multidisciplinary reassessment, leading to a coordinated strategy: PE/DPMAS for liver-directed support and CRRT for stage 3 AKI. Although the subsequent recovery was temporally associated with this approach, a single case cannot establish causality or determine the independent contribution of each modality.

## Data Availability

The original contributions presented in the study are included in the article/Supplementary Material, further inquiries can be directed to the corresponding authors.
